# Nanopore sequencing technology and its application in plant virus diagnostics

**DOI:** 10.3389/fmicb.2022.939666

**Published:** 2022-07-25

**Authors:** Kai Sun, Yi Liu, Xin Zhou, Chuanlin Yin, Pengjun Zhang, Qianqian Yang, Lingfeng Mao, Xuping Shentu, Xiaoping Yu

**Affiliations:** ^1^Zhejiang Provincial Key Laboratory of Biometrology and Inspection and Quarantine, College of Life Sciences, China Jiliang University, Hangzhou, China; ^2^Ausper Biopharma, Hangzhou, China; ^3^Hangzhou Baiyi Technology Co., Ltd., Hangzhou, China

**Keywords:** nanopore sequencing, NGS, virus detection, plant pathogens, virus quarantine

## Abstract

Plant viruses threaten crop yield and quality; thus, efficient and accurate pathogen diagnostics are critical for crop disease management and control. Recent advances in sequencing technology have revolutionized plant virus research. Metagenomics sequencing technology, represented by next-generation sequencing (NGS), has greatly enhanced the development of virus diagnostics research because of its high sensitivity, high throughput and non-sequence dependence. However, NGS-based virus identification protocols are limited by their high cost, labor intensiveness, and bulky equipment. In recent years, Oxford Nanopore Technologies and advances in third-generation sequencing technology have enabled direct, real-time sequencing of long DNA or RNA reads. Oxford Nanopore Technologies exhibit versatility in plant virus detection through their portable sequencers and flexible data analyses, thus are wildly used in plant virus surveillance, identification of new viruses, viral genome assembly, and evolution research. In this review, we discuss the applications of nanopore sequencing in plant virus diagnostics, as well as their limitations.

## Introduction

Because plant viruses cause devastating diseases and enormous economic losses worldwide in agricultural systems every year, they are major threats to sustainable and productive agriculture (Scholthof et al., 2011). Virus diseases are characterized by various symptoms, including ringspots, mosaic pattern development, leaf yellowing and distortion, plus impaired growth. However, diagnosis based on symptoms is unreliable because it lacks specificity. For example, some symptoms are quite subtle and can be easily confused with nutrient deficiencies and herbicide injury. Additionally, mixed infections often cause nonspecific and severe symptoms that further complicate virus identification. The expansion of international trade in agricultural products has accelerated the emergence and spread of plant viruses (Elena et al., 2014; Gilbertson et al., 2015). Therefore, rapid and effective detection of plant viruses is key to preventing and controlling infections.

Traditionally, plant viruses have been detected using morphological, PCR, and immunological analyses. However, these methods have several drawbacks, including long detection times, complicated preprocessing steps, and low detection efficiencies. Furthermore, these tests often require prior knowledge of viral genomic information or immunological characteristics that are not available for novel viruses.

Currently, metagenomic sequencing, represented by next-generation sequencing (NGS), has been widely used for detection and identification of plant viruses (Hadidi et al., 2016; Roossinck, 2017). NGS employs sequencing by synthesis, which determines the DNA sequences by capturing markers on newly-synthesized nucleotides (Goldberg et al., 2015). These methods do not require previous knowledge of viral sequences and can be used to sequence millions or billions of nucleotides in parallel. This permits detection of all viruses, including those not previously described (Mehetre et al., 2021). In theory, NGS can detect all viruses in a single assay and performance is only limited by the completeness of reference databases against which the sequences are compared. The resulting sequence information can also provide insight into virus population structure, ecology and evolution, as well as reveal virus variants that may contribute to various disease etiologies (Maree et al., 2018). However, NGS technology is not applicable for field detection of viruses, and although it can generate large datasets, the quality of sequencing needs improvement because the error rate of conventional NGS is about 1% and the length of sequence reads is generally short (35–700 bp)when compared with Sanger sequencing platforms (Fox et al., 2014; Kumar et al., 2019). Moreover, NGS sample preparation and actual sequencing are time-consuming and NGS equipment is expensive and often bulky (Gu et al., 2019), which limits widespread adoption for on-site pathogen detection or field deployment.

**Figure 1:**
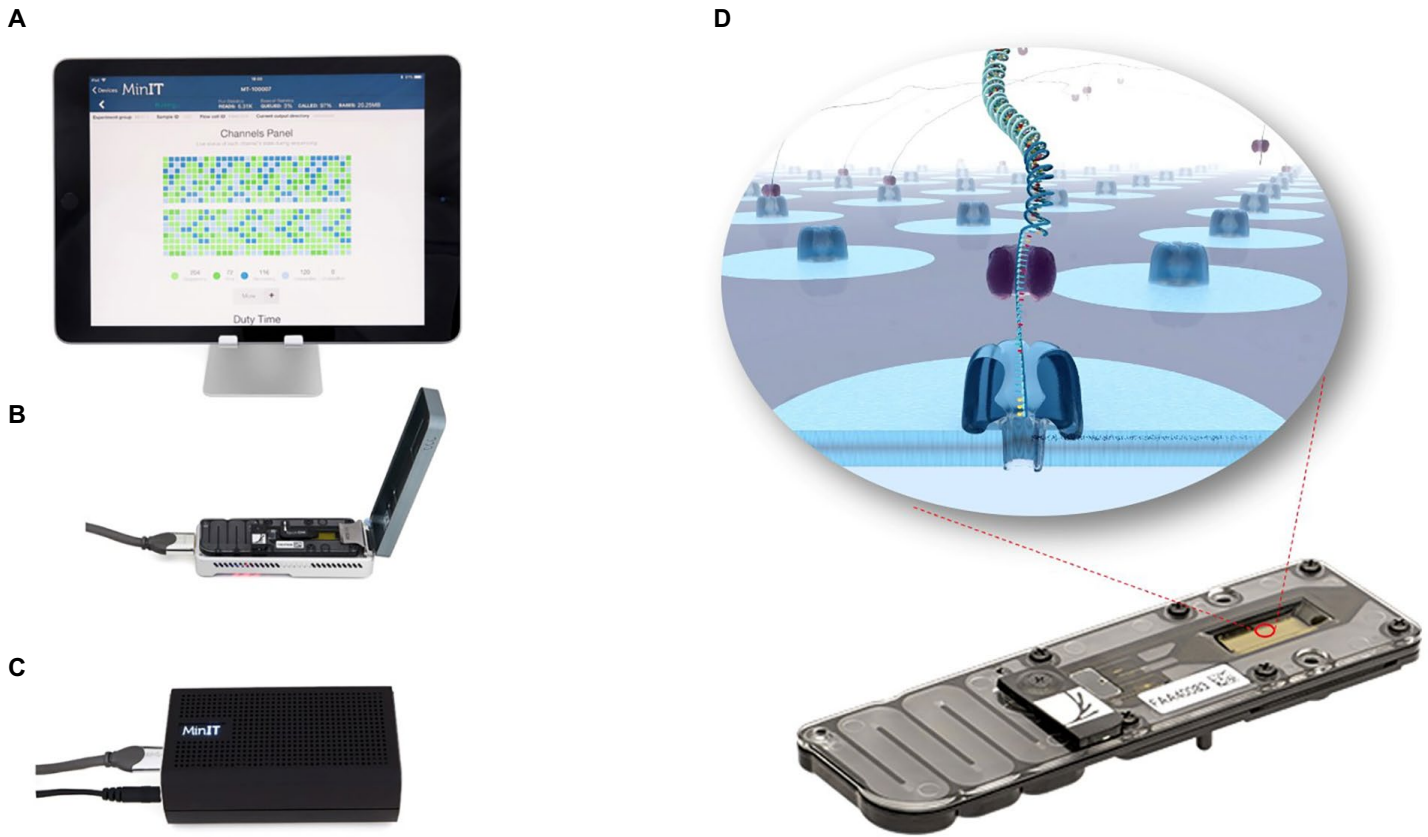
MinION sequencing device. MinION is a highly portable instrument (10  cm × 2  cm × 3.3  cm, about 90 g; **B**). It can be connected to an ONTs “MinIT” decoder **(C)** or to any computer with a USB port **(A)**. The entire nanopore sequencing process and real-time data analysis can be done on a PC. Nanopore sequencing is achieved by adding a sequencing library to the flowCell sequencing chip **(D)**. When DNA or RNA molecules pass through the nanopore, there is a shift in the nanopore current, which is measured by a sensor. The data is further transmitted and decoded into base signals that determine the nucleic acid sequence.

Nanopore sequencing, developed by Oxford Nanopore Technologies (ONT), is a third-generation sequencing technology that overcomes NGS technology shortcomings, by combining genetic engineering technology with computer based analyses (Lin et al., 2021). ONT nanopore sequencing has several advantages, including single-molecule sequencing, long sequencing read lengths, rapid sequencing speeds, and real-time monitoring of sequencing data (Laver et al., 2015; Deamer et al., 2016). Table 1 shows the performance of four high-throughput sequencing platforms. The most widely accepted nanopore sequencing platform is ONT MinION, a highly portable instrument (10 cm × 2 cm × 3.3 cm, 90 g), powered *via* a computer USB port (Figure 1). Unlike other sequencing technologies, sequencing and real-time data analysis can be completed on a personal computer. Coupled with its portability, nanopore technology has unprecedented applicability for plant virus detection. Here, we review the technical principles of nanopore sequencing and introduce applications for on-site, rapid, reliable, and sensitive plant virus detections.

**Table 1:** Comparison of the performance of four high-throughput sequencing platforms.

Sequencing platform	Sequencing principle	Average length	Merits	Faults
Illumina^a^	Sequencing by synthesis; fluorescently labeled dNTPs	≤300 bp	High accuracy	Short reads, high capital cost, time-consuming
Thermo Fisher’s Ion Torrent^a^	Sequencing by synthesis; detection of hydrogen ions	≤300 bp	High accuracy	Short reads, high capital cost, time-consuming
Pacific Biosciences^b^	Sequencing by synthesis; SMRTbell replication	3 kb	Long reads	High capital cost, variable accuracy, time-consuming
Oxford Nanopore^b^	Measure the changes in current as biological molecules pass through the nanopore	9 kb-10 kb	Long reads, low capital cost, portable	Low accuracy

a NGS platform.

b single molecular sequencing platform.

## Principle and advantages of nanopore sequencing technology

ONT nanopore sequencing is a promising, highly sensitive platform for identification of single strand DNA or RNA molecules. Unlike previous sequencing technologies, ONT nanopore sequencing identifies nucleotides based on electrical signals rather than optical signals (Deamer et al., 2016). This technology relies on a protein that forms a channel (nanopore) on a membrane. Upon application of an electrophoretic force, negatively charged biomolecules such as DNA or RNA pass through the nanopores and temporarily affecting the strength of the current flowing through the nanopore. The signal is then recorded and further analyzed to determine the nucleic acid sequence. The conversion of electrical signals into base sequences is called basecalling. It is the last step in the analysis of entire DNA/RNA sequences and uses deep learning to eliminate sequencing noise and signal errors. This step is crucial for improving sequencing accuracy and requires efficient algorithms and large datasets for computational training (Lin et al., 2021).

Nanopore sequencing allows direct DNA and RNA sequencing that does not require DNA synthesis reactions because nucleotides are recorded directly from the current signals. Whole-genome sequencing and methylation calling using Nanopore sequencing has been carried out with different species (Xu and Seki, 2020). For example, Kim et al. (2020) used direct RNA sequencing to obtain the COVID-19 genome sequence and detected at least 41 RNA modification sites on viral transcripts.

Nanopores can generate very long read lengths because the entire DNA or RNA fragment is analyzed. Thus, the read length directly correlates with the length of the DNA or RNA fragment. Users can choose appropriate nucleic acid preparation methods based on their experimental goals. Data released at the 2020 Nanopore scientific society conference showed that the longest sequence read lengths from nanopore sequencing have reached 4.1 Mb can be obtained from a single sequence read. Ultra-long read lengths are associated with higher quality and precision (Petersen et al., 2019). Nanopore sequencing permits real-time sequencing. Thus, the user can obtain data and experimental quality analysis as soon as sequencing begins, monitor the status of the sequencing, and stop the process as soon as sufficient data is available. Real-time sequence alignments can also be performed using the EPI2ME workflow, which enables rapid identification of pathogen-related sequences in the samples. Additionally, ONT tools^1^ are available to the user. These include Medaka, Tombo, Pomoxis, and Nanopolishare, which provide sequence correction, identification of modified nucleotides, genome assembly, and error correction in the genome assembly (Lu et al., 2016; Payne et al., 2019). Real-time Nanopore sequencing has a good application prospect in plant virus detection. Because early identification of viruses allows growers to take quick actions and effective sanitary measures. Real-time and on-site sequencing reduces overall costs and gives crop protection officers and farmers in rural communities’ information that is critical for sustainable crop production and management of pests and diseases (Boykin et al., 2019).

ONT provide a variety of sequencing platforms to meet various sequencing requirements, including MinION, a portable sequencer from ONT weighing about 100 g. The high throughput sequencers, GridION (which uses five MinION chips at a time) or PromethION (with 48 chips containing 3,000 nanopore channels each) allows for customization of chip number. Flongle, a one-off sequencing platform enables direct, real-time DNA or RNA sequencing and costs about $90, compared with the $900 MinION sequencing chip, and can generate about 1 GB of data in 24 h (Petersen et al., 2019). Thus, Flongle is a cost-effective and rapid option for smaller sequencing experiments.

The portability and real-time sequencing advantages of MinION provide an ideal platform for field sequencing because the method can provide results within 24 h of receiving samples with sequencing taking 15 to 60 min. In 2015, (Quick et al., 2016). shipped a nanopore sequencer in standard airline luggage to Guinea and conducted real-time monitoring of the West African Ebola outbreak. In a different trial, Boykin et al. (2019) detected cassava mosaic virus infections through metagenomics sequencing of cassava leaf, stem, and tuber samples using MinION at a cassava farm in the south of the Sahara desert. The whole process (collection of infected plant samples, nucleic acid database construction and sequencing analyses) was completed within 3 h after the researchers arrived at the farm.

## Methods for plant virus detection

In general, nanopore sequencing involves wet and dry lab steps. Wet lab work includes nucleic acid extraction and sequencing library preparation, which may take several hours. Dry lab work includes bioinformatics analysis, which can be completed within hours or days depending on the amount of data to be processed.

### The wet lab process

**Figure 2:**
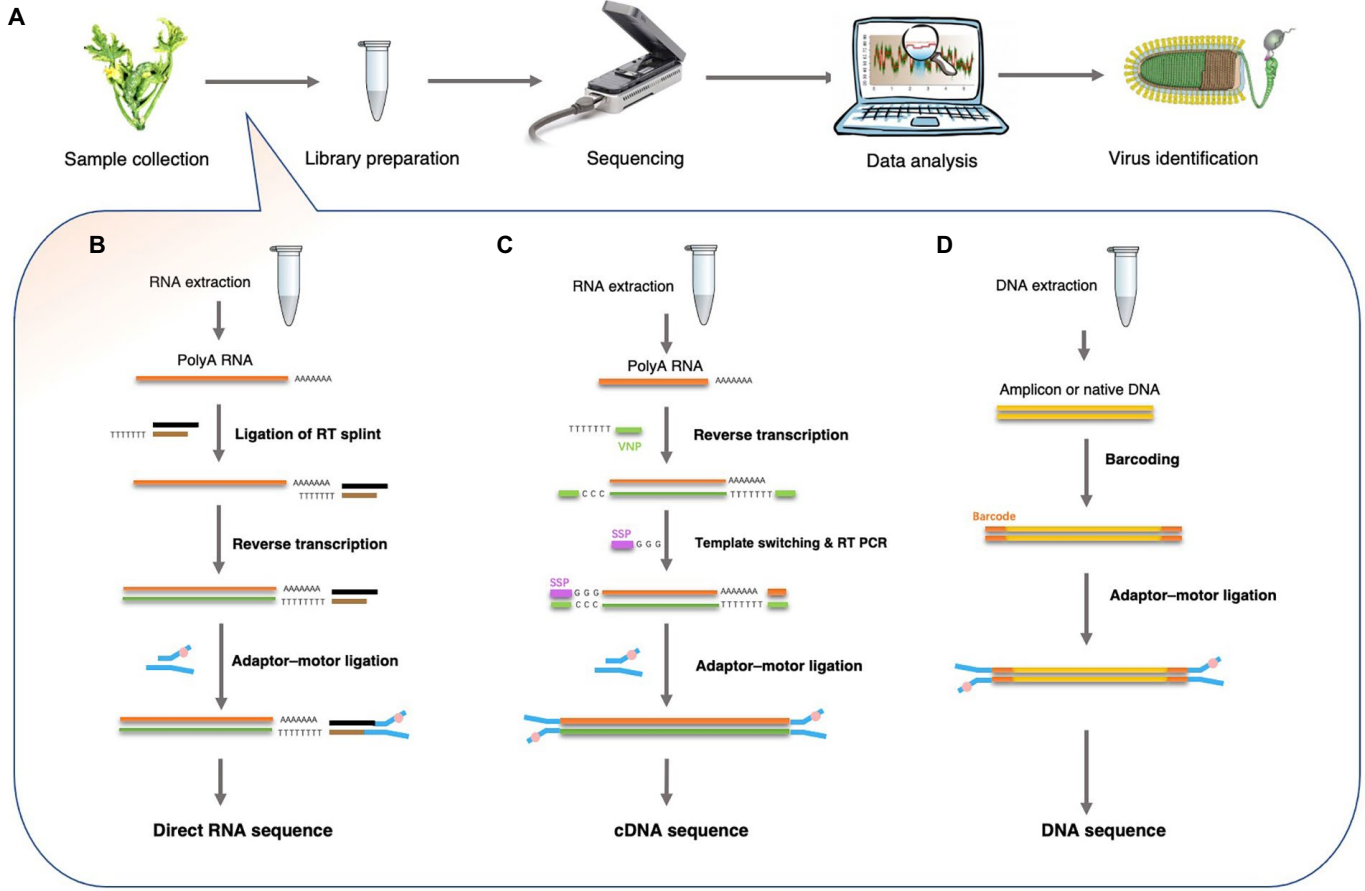
Overview of Nanopore sequencing and library preparation for plant virus detection. **(A)** Workflow for plant virus detection from sample collection to virus identification. **(B)** Direct RNA sequencing using the ONT-SQK-RNA001 library method with poly (A) RNA as a template. A reverse transcription step with oligo-dT primers was used to circumvent secondary structure of the RNA. A sequencing adapter was ligated to the mRNA using T4 DNA ligase. Since only the RNA strand is motor-ligated, only the RNA molecule is sequenced. **(C)** cDNA-PCR sequencing using the ONT-SQK-PCS108 library preparation method. Poly (A) RNA was used as a template for first strand cDNA synthesis with oligo-dT30VN primers (VNP). When first strand cDNA synthesis reaches the end of the RNA molecule, few non-templated Cs were added to the end of the cDNA by the reverse transcriptase. Then, a strand-switching primer (SSP) present in the reaction binds to the non-templated Cs, followed by enrichment. **(D)** DNA sequencing: the ONT-SQK-LSK108 library preparation method. The Barcoding Kit such as ONT-EXP-NBD103 can be used to tag the native DNA or amplicons DNA (after PCR or RCA reactions).

Viral nucleic acids, including genomic DNA or RNA, transcripts, and replicative intermediates can be noticed by a nanopore sequencer. For plant DNA viruses, several library preparation strategies have been established by ONT. For example, DNA from plant extracts can be directly sequenced using a ligation sequencing kit (SQK-LSK109) or amplified using PCR to obtain sufficient amplicons using a rapid barcoding kit (SQK-RBK004). Plant DNA viruses, both the ssDNA geminiviruses and the reverse-transcribing pararetroviruses, have circular genomes. Researchers often use roll-circle amplification (RCA) to enrich virus-derived DNA fragments and then break the circles into linear DNA strands before sequencing on a nanopore sequencing platform (Figure 2).

However, nanopore methods that detect DNA cannot detect RNA viruses directly. Thus, RNA is directly sequenced or first converted into cDNA before being used to detect plant viruses. Current ONT library preparation kits include a direct cDNA sequencing kit (SQK-DCS109), a PCR-cDNA sequencing kit, with (SQK-PCB109) and without (SQK-PCS109) barcoding, and a direct RNA sequencing (SQK-RNA002) kit (Liefting et al., 2021). These kits are designed for polyadenylated [poly(A)] RNA samples (Figure 2). Hence, plant viruses that lack poly(A) tails, cannot be sequenced using these strategies. However, multiple strategies have been developed for sequencing RNA that lack poly (A) tails. In one strategy, a poly (A)-tailing reaction is carried out on the total RNA using *E. coli* poly (A) polymerase and then using the resulting poly (A) RNA as input for the ONT direct cDNA sequencing kit (SQK-DCS109). In another strategy, double-stranded (ds) cDNA is synthesized using random hexamers and used as input in the end-prep step of the direct cDNA sequencing kit (SQK-DCS109). These methods have proven to be efficient in viral metagenomics and enable the use of nanopore sequencing to detect all types of plant viruses (Palanga et al., 2016; Claverie et al., 2018).

### The dry-lab process

**Figure 3:**
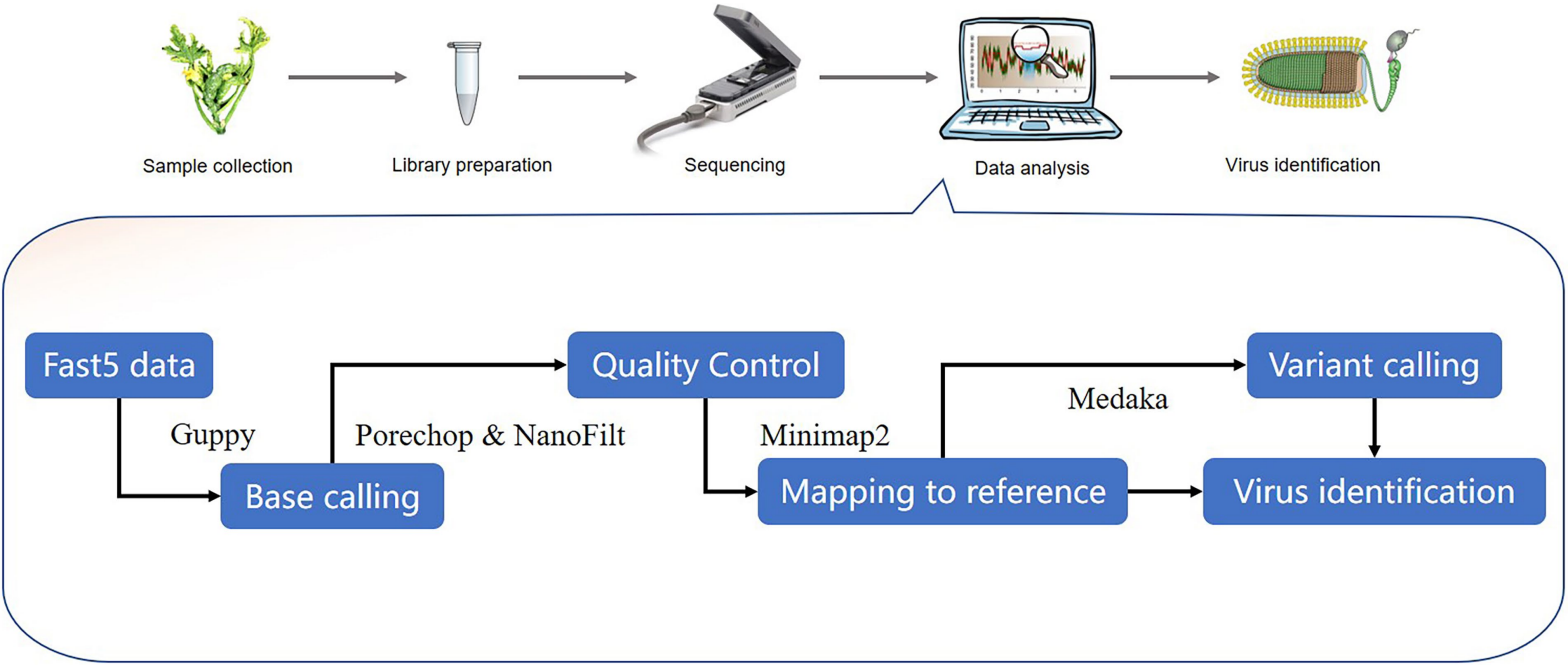
Outline of potential stages in the workflow for nanopore-seq analysis for plant virus detection. Representative bioinformatics tools are shown in the workflow.

A number of bioinformatics tools have been used for virus sequence analysis and diagnosis. The virus detection workflow needs to: (1) parse input electropherogram files to obtain base sequences, (2) conduct quality control measures on raw data files, including trimming of poor quality reads and adaptor sequences, and (3) assemble and map reads in order to identify known and novel viruses (Figure 3). Recent software for the analysis of nanopore data are shown on Table 2.

**Table 2:** Bioinformatics tools for the identification of plant viruses using nanopore sequencing.

Software	Reference/introduction page	Functions	Availability
MinKNOW	/	MinKNOW is Oxford Nanopore Technologies Device Control software. Core tasks: data acquisition, real-time analysis and feedback, basecalling, data streaming, device control (including selecting the run parameters), sample identification and tracking, and ensuring that the platform chemistry is performing correctly to run the samples.	https://nanoporetech.com
BLAST+	Camacho et al. (2009)	BLAST command-line applications, compared to the current BLAST tools it substantially improves the speed for long queries and chromosome length database sequences	https://ftp.ncbi.nlm.nih.gov/blast/executables/blast+/LATEST/2022-03-14
seqmagick	/	Seqmagick is a kickass little utility built in the spirit of imagemagick used to expose the file format conversion in Biopython in a convenient way	https://fhcrc.github.io/seqmagick/
seqtk	/	Seqtk is a fast and lightweight tool for processing sequences in the FASTA or FASTQ format. It seamlessly parses both FASTA and FASTQ files which can also be optionally compressed by gzip.	https://github.com/lh3/seqtk
minimap2	Li (2018)	Minimap2 is a general-purpose alignment program to map DNA or long mRNA sequences against a large reference database. It performs split-read alignment, employs concave gap cost for long insertions and deletions, and introduces new heuristics to reduce spurious alignments.	https://github.com/lh3/minimap2
samtools	Li et al. (2009)	Samtools is a set of utilities that manipulate alignments in the SAM (Sequence Alignment/Map), BAM, and CRAM formats. It converts between the formats, does sorting, merging and indexing, and can retrieve reads in any regions swiftly.	https://github.com/samtools/
EFetch	https://www.ncbi.nlm.nih.gov/books/NBK25499/#chapter4.EFetch	Returns formatted data records for a list of input UIDs Returns formatted data records for a set of UIDs stored on the Entrez History server	https://eutils.ncbi.nlm.nih.gov/entrez/eutils/efetch.fcgi
poRe	Watson et al. (2015)	poRe, a package for R that enables users to manipulate, organize, summarize and visualize MinION nanopore sequencing data.	http://sourceforge.net/projects/rpore/
Canu	Koren et al. (2017)	Canu is a fork of the Celera Assembler designed for high-noise single-molecule sequencing (such as the PacBio RSII or Oxford Nanopore MinION).	https://github.com/marbl/canu/
Medaka	/	medaka is a tool for creating consensus sequences and variant calls from nanopore sequencing data.	https://github.com/nanoporetech/medaka
iVar	Grubaugh et al. (2019)	iVar generates virus consensus genomes from sequencing data across multiple replicates	https://github.com/andersen-lab/ivar
porechop	/	Porechop is used to remove nanopore adapters and chimeric reads.	https://github.com/rrwick/Porechop
NanoFilt	De Coster et al. (2018)	Filtering on quality and/or read length, and optional trimming after passing filters.	https://github.com/wdecoster/nanofilt
EPI2ME	/	EPI2ME is a cloud-based data analysis platform, offering easy access to several workflows for end-to-end analysis of nanopore data in real-time. An intuitive graphical interface facilitates the interpretation of individual or multiple barcoded samples.	https://epi2me.nanoporetech.com/
Prowler	Lee et al. (2021)	A Trimming and filtering tool for Nanopre sequencing analysis	https://github.com/ProwlerForNanopore/ ProwlerTrimmer.

## Examples of plant virus detection using nanopore sequencing

Although Nanopore sequencing technology is relatively new, several studies have used the technology to detect plant viruses (Table 3).

**Table 3:** Summery of viruses detected in plants using nanopore sequencing technology.

Host	Sequencing library type	Library preparing kit	Virus	Genome type	References
Peach	WTA^a^-DNA	ligation sequencing kit (SQK-MAP006)	PPV	ssRNA(+) with poly (A) tract	Bronzato Badial et al. (2018)
cassava	DNA directly	Rapid Barcoding kit (SQK-RBK004)	ACMV	ssDNA	Boykin et al. (2019)
	DNA directly	Rapid Barcoding kit (SQK-RBK004)	EACMV	ssDNA	Boykin et al. (2019)
	RCA^b^-DNA	ligation sequencing kit (SQK-LSK109)	SLCMV	ssDNA	Leiva et al. (2020)
	cDNA directly	Direct cDNA Sequencing Kit (SQK-DCS109)	Cassava torrado-like virus	ssRNA(+) with poly (A) tract	Leiva et al. (2022)
Yam	cDNA-PCR-DNA	cDNA-PCR kit (SQK-PCS108)	DBV	dsDNA	Filloux et al. (2018)
	cDNA-PCR-DNA	cDNA-PCR kit (SQK-PCS108)	YMMV	ssRNA(+) with poly (A) tract	Filloux et al. (2018)
	cDNA-PCR-DNA	cDNA-PCR kit (SQK-PCS108)	YCNV	ssRNA(+) with poly (A) tract	Filloux et al. (2018)
Cowpea	RCA^b^-DNA	ligation sequencing kit 1D ^c^	CoBYMV	ssDNA	Naito et al. (2019)
Wheat	cDNA-PCR-DNA	cDNA sequencing kit (SQK-PCS108)	WSMV	ssRNA(+) with poly (A) tract	Fellers et al. (2019)
	cDNA-PCR-DNA	cDNA sequencing kit (SQK-PCS108)	TriMV	ssRNA(+) with poly (A) tract	Fellers et al. (2019)
	cDNA-PCR-DNA	cDNA sequencing kit (SQK-PCS108)	HPWoMV	segmented RNA(−) lack poly (A) tract	Fellers et al. (2019)
Tomato	DNA directly	Sequencing Kit 1D (SQK-LSK 108)	TYLCV	ssDNA	Chalupowicz et al. (2019)
	RNA directly	direct RNA sequencing protocol^c^	TBRFV	ssRNA(+) lack poly (A) tract	Chalupowicz et al. (2019)
	RCA^b^-DNA	Ligation Sequencing Kit (SQK-LSK109)	tomato mottle leaf distortion virus	ssDNA	Martins et al. (2021)
watermelon	DNA directly	Sequencing Kit 1D (SQK-LSK 108)	WCSV	ssDNA	Chalupowicz et al. (2019)
watermelon	RNA directly	direct RNA sequencing protocol^c^	CGMMV	ssRNA(+) lack poly (A) tract	Chalupowicz et al. (2019)
squash	RNA directly	direct RNA sequencing protocol^c^	ZYMV	ssRNA(+) with poly (A) tract	Chalupowicz et al. (2019)
potato	cDNA-PCR-DNA	Ligation Sequencing Kit (SQK-LSK109)	Potato virus Y	ssRNA(+) with poly (A) tract	Della Bartola et al. (2020)
Medicago	RCA^b^-DNA	PCR Barcoding Kit (SQK-PBK004)	capulavirus Trifolium virus 1	ssDNA	Ben Chehida et al. (2021)
	PCR-DNA	PCR-cDNA Sequencing Kit (SQK-PCS109)	capulavirus Trifolium virus 1	ssDNA	Ben Chehida et al. (2021)
Jasmine	cDNA-PCR-DNA	cDNA-PCR sequencing kit (SQK-PCB109)	jasmine virus C	ssRNA(+) with poly (A) tract	Amoia et al. (2022)

a WTA: whole transcriptome amplification, total RNA content was amplified using the TransPlex WTA Kit (Sigma Aldrich, St. Louis, MO).

b RCA: rolling circle amplification, DNA was consequently amplified by RCA using Phi29 DNA polymerase (New England Biolabs, United States).

c Product code is not provided in this research.

### Peach (*Prunus persica*)

Through whole transcriptome analysis with the Oxford MinION technology, plum pox virus (PPV) was detected in *P. persica* (peach; Bronzato Badial et al., 2018). Briefly, total RNA was extracted from plant samples and amplified using a TransPlex WTA kit (Sigma Aldrich, St. Louis, MO). A cDNA library was then prepared and loaded into the MinION flow cell and sequenced for 24 h and the raw electrical data was processed with Metrichor (Oxford Nanopore). FASTA sequences and read event data, including strand translocation times, were extracted and analyzed by the R package, poRe (Watson et al., 2015). The GraphMap Aligner was used for nucleotide base alignment (Sović et al., 2016) and the resulting contigs were compared with the complete set of high quality, annotated, viral genomes on NCBI RefSeq.^2^ This method permitted the simultaneous detection of PPV in peach plant after a 38 s run, demonstrating the capability of this methodology to obtain results rapidly. Results showed that this methodology is useful for detecting unsuspected viral pathogens in plant.

### Cassava (*Manihot manifera*)

Two studies Nanopore sequencing have investigated virus infections in cassava. Boykin et al. (2019) combined PDQeX DNA purification technology with the MinION and MinIT mobile sequencing devices to generate an effective point-of-need field diagnostic system with cassava samples from Tanzania, Uganda, and Kenya. Barcoded DNA libraries were generated with the rapid barcoding kit, SQK-RBK004 DNA sequencing was conducted with a MinION connected to MinIT, a portable live base calling server. Reads across all of the sequencing runs (mean read length: 35–948 bp) were compared with a cassava mosaic disease reference database using BLASTn. The study detected several viruses in real time, including African cassava mosaic virus (ACMV) and the East African cassava mosaic virus (EACMV). This case study was designed to show the possibility to go from sample to diagnosis, in a regional setting, on farm in 3 h versus the normal 6 months with conventional methods. The results of this research show that it is possible to use a range of battery powered devices to achieve DNA extraction, long read sequencing and analysis all under a tree on the farm while the farmers wait for results (Boykin et al., 2019). In the other study, nanopore sequencing detected Sri Lankan cassava mosaic virus (SLCMV) in cassava samples from Thailand (Leiva et al., 2020). In this case, total DNA was extracted from an infected cassava plant with cetyltrimethylammonium bromide and Phi29 DNA polymerase was used for RCA. The DNA library was prepared with a ligation sequencing kit (SQK-LSK109) and then sequenced in a FLO-MIN106D (R9.5) flow cell. The assembly was assembled from the raw reads *via de novo* assembly (using Canu v1.8) and reference assembly (using Minimap2 Li, 2018) and Pilon (Walker et al., 2014) with distinct SLCMV genome sequences. The resulting contigs contained 72,800 reads for DNA-A, with an average coverage of 15,000 X, and 70,681 reads for DNA-B, with an average coverage of 6,000 X. This study provides a well-arranged sequencing process for obtaining complete geminivirus genomes using nanopore technology.

### Yam (*Dioscoriaalata*)

Multiple virus infections have been identified in yams (*Dioscoriaalata*) with a nanopore cDNA-seq analysis (Filloux et al., 2018) in which a MinION sequencing library was prepared with the SQK-PCS108 cDNA-PCR kit (Oxford Nanopore Technologies Ltd). Briefly, cDNA was first generated from total RNA from an infected yam by revers transcription (strand-switching method) and then amplified by PCR. The PCR product was ligated with adapters, loaded onto a R9.4 Flow Cell (FLO-MIN106 R9.4) and sequenced for 48 h with MinION. The MinION reads were assembled *de novo* using Canu v1.6 (Koren et al., 2017). and compared against the GenBank database with DIAMOND (Buchfink et al., 2015). The study also compared the use of small RNA Illumina sequencing with nanopore sequencing for virus detection. The two methods detected three viruses, Dioscorea bacilliform virus (DBV), yam mild mosaic virus (YMMV) and yam chlorotic necrosis virus (YCNV). However, MinION sequencing failed to detect ampelovirus-related sequences, which were detected by Illumina. The study determined that this failure resulted from the fact that the Nanopore cDNA sequencing protocol used specifically targeted poly (A) sequences and hence missed viruses that lack 3′ poly (A) tails. Furthermore, this research shows that the consensus sequence obtained either by *de novo* assembly or after mapping the MinION reads on the virus genomic sequence was >99.8% identical with the Sanger-derived reference sequence (Filloux et al., 2018). These degrees of sequencing accuracy demonstrate that the Nanopore sequencing approach can be used to both reliably detect and accurately sequence nearly full-length virus genomes with positive-sense single-strand polyadenylated RNA.

### Cowpea (*Vigna unguiculata*)

Nanopore sequencing technology was used to identify a novel bipartite begomovirus in cowpea plants which has been designated cowpea bright yellow mosaic virus (CoBYMV; Naito et al., 2019). In this case, total DNA was extracted from cowpea samples exhibiting bright golden mosaic symptoms and subjected to RCA using Phi29 DNA polymerase. The DNA library was prepared with the ligation kit 1D and sequenced with a Spot-on flowcell (FLO-MIN106) on the MinION Mk1B. The raw FAST5 reads were uploaded to the online server for base calling through the Metrichor EPI2ME platform. The base-called MinION reads were converted to FASTQ and assembled using Canu v1.7 (Koren et al., 2017). The resulting contigs were then compared against the viral protein RefSeq database using Blastx in Geneious version 9.1.2 (Kearse et al., 2012). The assembled contigs of the putative complete DNA-A and DNA-B sequences were confirmed by PCR and Sanger sequencing. The MinION derived consensus sequences had 100% nucleotide sequence identity with the corresponding Sanger sequences, except for one nucleotide in a T-rich region of DNA B. This study also demonstrated that a portable nanopore sequencing device is a rapid and accurate alternative tool for the characterization of novel plant virus.

### Wheat (*Triticum aestivum*)

Wheat streak mosaic virus (WSMV), triticum mosaic virus (TriMV), and high plains wheat mosaic virus (HPWoMV) together comprise the wheat streak mosaic complex (WSM), which causes losses of up to 5% of U.S (Fellers et al., 2019). wheat production. Use of resistant wheat cultivars consisting primarily of introgressed *Wsm1* and *Wsm2* resistance genes is the most economical and effective method for disease control. However, in 2015, the ‘Clara CL’ resistant variety harboring *Wsm2* resistance developed severe mosaic symptoms and stunted growth in Hamilton County, KS (Fellers et al., 2019). To investigate virus variants that could circumvent the *Wsm2* resistance, infected wheat tissue was sequenced by nanopore sequencing with the 1D cDNA sequencing kit (SQK-LSK108) in two cDNA reactions, one of which used XhoI-oligo-d(T)20 for poly (A) viruses and the other used random 6-mer primers for poly (A) minus RNA viruses. A DNA library was prepared from 500 ng of amplified cDNA and the samples were loaded onto a MinION 107 v9.5 Flow Cell. Raw data base-calling and adapter trimming was carried out by MinKNOW, with albacore v1.7.3. and porechop v0.2.3 (Wick, R, University of Melbourne). Clean data was then aligned to a cereal virus reference file with CLC Genomics Workbench v11 (Qiagen). Non-wheat virus assemblies were compared against the NCBI’s virus genome database by BLAST analyses, which revealed the presence of mixed infections of WSMV, TriMV and BYDV-PAV. The study also revealed that susceptibility resulted from a nucleotide sequence difference in the WSMV field isolates at position 6,833 that resulted in threonine (T) substitutions for valine (V) or methionine (M). One or more of these differences may explain why resistant wheat cultivars exhibited WSMV symptoms. These results demonstrate that ONT can more accurately identify causal virus agents and has sufficient resolution to provide evidence of causal variants.

### Tomato (*Solanum lycopersicum*), cucumber (*Cucumis sativus*) and butternut squash (*Cucurbita moschata*)

To evaluate Nanopore sequencing for diagnosis of plant diseases, a recent study described DNA or RNA sequencing of symptomatic plant tissues infected with known viruses (tomato with tomato yellow leaf curl virus or tomato brown rugose fruit virus, watermelon samples with watermelon chlorotic stunt virus or an unknown virus that elicited yellowish leaves and mottling, and butternut squash with mosaic leaf symptoms; Chalupowicz et al., 2019). A DNA library was prepared from nucleic acid extracted from these plants with the ligation sequencing kit 1D (SQK-LSK 108). Direct RNA library preparations were carried out with the Oxford Nanopore direct RNA sequencing protocol. The sequences were run on the MinION flow cell (FLO-Min106 version R9.4, Oxford Nanopore Technologies) and assembled on the MinION sequencer (MK 1B version, Oxford Nanopore Technologies). MinION basecalling was conducted for 6 h in real-time on a local computer with MinKNOW 2.0 version 18.03.1 software (Oxford Nanopore Technologies Ltd.). The basecalled reads were then uploaded by use of EPI2ME desktop agent software (version 2.52.1202033). The “What’s in my Pot?” (WIMP) workflow was used to assign taxonomic classifications of the MinION reads based on the NCBI RefSeq virus database.^3^ DNA viruses were identified within the DNA sequencing samples, and the CGMMV and ZYMV RNA viruses were detected during RNA sequencing of unknown samples. These findings highlight the ability of the Nanopore platform to detect viruses through direct DNA or RNA sequencing.

## Summary and future prospective

Nanopore sequencing technology allows direct sequencing of DNA or RNA samples and provides fast and real-time dynamic monitoring of sequencing data in the field. However, compared with plant bacterial and fungal pathogens, the detection of plant viruses using nanopore sequencing faces greater challenges. As we all known that the genomic meterials of fungi and bacteria are DNA. In the detection of plant bacteria and fungi, DNA direct sequencing or marker gene DNA amplification sequencing method is mainly used. The most commonly used marker genes in metataxonomic are the 16S rRNA for bacteria and archaea, and the 18S rRNA for fungi, while there are no marker genes for viruses (Ciuffreda et al., 2021). In addition, viruses have more diverse genome types classified into DNA viruses and RNA viruses. Furthermore, RNA genomes can be divided into those carrying Poly (A) tails and those without Poly(A) tails. There is currently no general library construction method to achieve sequencing of all types of plant viruses using nanopore sequencing technology. So, researchers must choose a suitable library construction strategy according to the characteristics of target viruses.

To improve field detection efficiency, pre-screened databases that only contain data of the suspected disease and host genome information are often used (Boykin et al., 2019). Consequently, existing or new field viruses may not be identified depending on the status of the databases until subsequent data analysis when the scientist has returned to the lab or is within range of a good internet connection capable of uploading large amounts of data to the cloud. Nonetheless, the current nanopore sequencing technology is the most economical and convenient sequencing platform for detection of plant viruses in the field. However, the use of nanopore sequencing to detect human pathogens is at a more advanced stage compared with plant viruses. Therefore, we have discussed the latest application of nanopore sequencing technology in clinical diagnosis of pathogens with a view towards providing information to improve the detection of plant viruses.

The combination of nanopore sequencing technology and isothermal amplification technology has been shown to shorten detection times and improve detection efficiency. Boykin et al. (2019) developed a simple and accurate molecular diagnosis method combining Loop-mediated isothermal amplification (LAMP) and nanopore sequencing for detection of malaria (Imai et al., 2017). This protocol included 18S rRNA specific LAMP primers for human plasmodium and tested blood samples from 63 malaria patients. The LAMP product was sequenced using MinION, which revealed that the sequence obtained was consistent with that of the reference plasmid sequence and nested PCR results. Bi et al. (2021) developed a method for multiplex isothermal amplification-based sequencing and real-time analysis of multiple viral genomes, referred to as the nanopore sequencing of isothermal rapid viral amplification for near real-time analysis. The design of the method provided the ability to simultaneously detect SARS-CoV-2, influenza A, human adenovirus, and human coronavirus, as well as to identify mutations in up to 96 samples in real time. These methods can avoid the false positives caused by isothermal amplification, and also increase the content of targeted nucleic acids in the sequencing library.

The application of bioinformatics tools in field applications has continuously improved sequencing accuracy, read length, and throughput. For example, a new software named UNCALLED developed by Kovaka et al. (2021) has improved the sequencing of target nucleic acid amounts by manipulating the nanopore current. The software has the potential to quickly match the nanopore current signal flow to a preset sequence reference database. This allows matching targeted nucleic acids to pass through the nanopore to obtain the sequences of full-length molecules. In cases where the sequence does not match the reference sequence, the software will reverse the voltage across the nanopore and physically eject the nucleic acid molecules to create room for the next nucleic acid. In the study, UNCALLED was employed to eliminate the sequence information of known bacterial genomes in metagenomic populations, in order to enrich the nucleic acids of other species by nearly five folds. A total of 148 human genes associated with hereditary cancer were enriched by nearly 30 folds, and the numbers of single-nucleotide polymorphisms, insertions, and deletions, structural variations, and methylation variants in the analyses were more than twice the number of those detected by the 50X coverage in short read-lengths of the whole genome sequencing.

High-throughput sequencing can be used to characterize virus evolution and to identify adaptations that affect transmission or pathogenicity. Coupled with a high replication rate, plant RNA viruses can form a virus population containing a group of genetically related but different haplotypes. Many attempts have been made to reconstruct viral haplotypes using NGS reads. However, the short length of NGS reads cannot cover distant single-nucleotide variants, making it difficult to reconstruct complete or near-complete haplotypes (Cai and Sun, 2022). Long-read ONT data can be used to study sample-specific genomic details, including structural variation (SV) and haplotypes. Given that single long reads can encompass multiple variants, including both single-nucleotide variants (SNVs) and SVs, it is possible to perform phasing of haplotype-resolved analyses with appropriate bioinformatics software, such as LongShot for SNV detection and WhatsHap for haplotyping/phasing (Wang et al., 2021).

Although single-nucleotide variations (SNVs) are considered to be key drivers of virus adaptation, RNA recombination events that delete or insert nucleic acid sequences have also been implicated in this process. Jaworski et al. (2021) designed an approach called ‘Tiled-ClickSeq’ to provide complete genome coverage, including the 5′UTR, at high resolution and virus specificity on both Illumina and Nanopore platforms. Using the developed method, these platforms simultaneously analyzed multiple SARS-CoV-2 isolates and clinical samples and characterized minority variants, sub-genomic mRNAs, structural variants, and D-RNAs. This indicates that the Tiled-ClickSeq is a convenient and robust platform for SARS-CoV-2 genomics because it captures a full range of RNA species in a single and simple assay. Using the nanopore long-read sequencing platform, Westergren and Colleagues revealed that the complex human adenovirus type 2 (Ad2) transcriptome has a flexible splicing machinery that generates several mRNAs from the early and late transcription units (Westergren Jakobsson et al., 2020). More than 900 alternatively spliced mRNAs generated from the Ad2 transcriptome were identified among which 850 were novel mRNAs. The nanopore sequencing provides access to genomic regions that cannot be accessed by traditional sequencing methods. Thus, application of the nanopore technology for detection and analyses of plant virus can reveal structural variations and can promote early diagnosis, treatment, and monitoring of plant viruses.

Nanopore sequencing technology provides a very convenient method for investigating the role of epigenetics in the regulation of plant virus activities. Epigenetic covalent nucleotide modifications including 5-methylcytosine (5-mC), N4-methylcytosine (4-mC), and N6-methyladenine (6-mA), which do not alter the primary DNA/RNA sequence, participate in the regulation of virus replication. Long-read nanopore sequencing enables direct observation of modified nucleotides by assessing deviating current signals as has been revealed by findings from animal viruses studies. Goldsmith et al. (2021) proposed a nanopore sequencing method for detection of 5mCpG on the HBV genome, which does not rely on bisulfite conversion or PCR. They found that assessment of 5mCpG levels in HBV by bisulfite-quantitative methyl-specific qPCR and nanopore sequencing were highly correlated in their ~2000 times coverage of the viral genome. Zhang et al. (2021) conducted methylated RNA immunoprecipitation sequencing and nanopore direct RNA sequencing analyses to show that SARS-CoV-2 RNA contained m6A modifications. Moreover, the results indicated that SARS-CoV-2 infection not only increased the expression of methyl transferase-like 3 (METTL3) but also altered the distribution of the protein, and these modifications of METTL3 expression affected virus replication. Collectively, the findings described above demonstrate that nanopore technology can effectively detect nucleotide modifications during viral infection.

Currently, the biggest obstacle to application of nanopore sequencing technology is its comparatively lower read accuracy when compared to short read sequencers. Nanopore sequencing platforms have sequencing accuracy rates between 97 and 99%, depending on the flow cell chemistry and the mode of basecalling step. In recent years, ONT has improved the read quality of nanopore sequencing by changing the chemical reagents used in library preparation kits and flow cells and developing improved algorithms for basecalling (Javaran et al., 2021). For example, higher raw read accuracy (98.3%) was achieved by a new basecaller software, Bonito (Silvestre-Ryan and Holmes, 2021). Additional improvements include sequencing kits based on a new chemistry, Q20+, which has been tested in nanopore flow cells and will be released in the near future. This technology uses a refined motor protein (E8.1), which increases the raw read accuracy to 99.3%.^4^ Bioinformatic analysis has significant impact to the accuracy of Nanopore sequencing. Different computational pipelines of the same nanopore data may lead to different results. Normally, MinION pipeline contains primer trimming, alignment, variant calling and consensus generation (Yang et al., 2020). For example, there are multiple updates to the Guppy algorithm in recent years. At the London Calling meeting of 2020, the median single read accuracy for Guppy 3.6.0 when sequencing a mixture of reference microbial genomes or the human genome was reported at 96.5%. While the current version of Guppy (6.1.1) released in March 2022 increased the sequencing accuracy to 99.2%.^5^ If the current raw reading accuracy is as claimed by Oxford Nanopore Technologies, we think the remaining errors do not have a great impact on virus identification and this technology is still an interesting option for virus detection from a variety of biological samples.

Another major hindrance to the application of nanopore sequencing is the lack of a bioinformatics system that is fast, accurate, and easy to use. Although many bioinformatics tools have been developed for nanopore sequencing, their adaptability, accuracy, robustness, and efficiency are far from being satisfactory. In typical use cases, bioinformatics software requires users who are proficient in using commands and codes to perform analysis operations in the Linux environment. A webserver is the best option for non-expert users, but this requires strong computer hardware at the remote site and is associated with difficulties in uploading large files.

Taken together, these results indicate that the nanopore sequencing technologies have high diagnostic efficiency for plant viruses and can enrich pathogenic resources in plant disease databases. In the future, these technologies are expected to provide accurate, rapid, and on-site diagnoses for numerous phytosanitary requirements.

## Author contributions

YL and KS: writing—original draft preparation. CY and PZ: writing—review and editing. XY: project administration. KS and XS: funding acquisition. All authors contributed to the article and approved the submitted version.

## Funding

This research was supported by Key Research Program of Zhejiang Province of China (No. 2022C02047); National Natural Science Foundation of China (No. 32102165); Zhejiang Provincial Natural Science Foundation (No. LQ22C140003); and Zhejiang Province Natural Science Foundation of China (No. LGC21C140001).

## Conflict of interest

XZ was employed by the company Ausper Biopharma. LM was employed by the company Hangzhou Baiyi Technology Co., Ltd.

The remaining authors declare that the research was conducted in the absence of any commercial or financial relationships that could be construed as a potential conflict of interest.

## Publisher’s note

All claims expressed in this article are solely those of the authors and do not necessarily represent those of their affiliated organizations, or those of the publisher, the editors and the reviewers. Any product that may be evaluated in this article, or claim that may be made by its manufacturer, is not guaranteed or endorsed by the publisher.

## Footnotes

^1^ https://github.com/nanoporetech

^2^ ftp://ftp.ncbi.nlm.nih.gov/refseq/release/viral

^3^ ftp://ftp.ncbi.nlm.nih.gov/genomes/refseq/

^4^ https://nanoporetech.com/q20plus-chemistry

^5^ https://community.nanoporetech.com/posts/guppy-v6-1-1-release
